# Simvastatin-Induced Insulin Resistance May Be Linked to Decreased Lipid Uptake and Lipid Synthesis in Human Skeletal Muscle: the LIFESTAT Study

**DOI:** 10.1155/2018/9257874

**Published:** 2018-09-12

**Authors:** Steen Larsen, Andreas Vigelsø, Sune Dandanell, Clara Prats, Flemming Dela, Jørn Wulff Helge

**Affiliations:** ^1^Xlab, Center for Healthy Aging, Department of Biomedical Sciences, Faculty of Health Sciences, University of Copenhagen, Copenhagen, Denmark; ^2^Clinical Research Centre, Medical University of Bialystok, Bialystok, Poland; ^3^Department of Physiotherapy and Occupational Therapy, Metropolitan University College, Copenhagen, Denmark; ^4^Department of Geriatrics, Bispebjerg University Hospital, Copenhagen, Denmark

## Abstract

**Background:**

A prevalent side-effect of simvastatin is attenuated glucose homeostasis. The underlying mechanism is unknown, but impaired lipid metabolism may provide the link. The aim of this study was to investigate whether simvastatin-treated patients had a lower capacity to oxidize lipids and reduced expression of the major proteins regulating lipid uptake, synthesis, lipolysis, and storage in skeletal muscle than matched controls.

**Materials and Methods:**

Ten men were treated with simvastatin (HbA1c: 5.7 ± 0.1%), and 10 healthy men (HbA1c: 5.2 ± 0.1%) underwent an oral glucose tolerance test and a muscle biopsy was obtained. Fat oxidation rates were measured at rest and during exercise. Western blotting was used to assess protein content.

**Results:**

Patients treated with simvastatin had impaired glucose tolerance compared with control subjects, but fat oxidation at rest and during exercise was compatible. Skeletal muscle protein content of CD36, lipoprotein lipase (LPL), and diacylglycerol acyltransferase (DGAT) 1 were lower, and DGAT 2 tended to be lower in patients treated with simvastatin.

**Conclusions:**

Patients treated with simvastatin had a reduced capacity to synthesize FA and diacylglycerol (DAG) into triacylglycerol in skeletal muscle compared to matched controls. Decreased lipid synthesis capacity may lead to accumulation of lipotoxic intermediates (FA and DAG) and hence impair glucose tolerance.

## 1. Introduction

The family of HMG-Co A reductase inhibitors, known as statins, is widely used in the treatment of hypercholesterolemia [1]. Unfortunately, the widely used simvastatin has been associated with attenuated insulin sensitivity [2, 3], but the underlying mechanisms remain unknown. However, Phillips et al. have observed larger lipid droplets in skeletal muscle from humans in statin treatment [4]. Furthermore, Langhi et al. recently used mouse and human primary hepatocytes to link statins to altered lipid droplet regulation [5]. One mechanism could therefore be altered muscle lipid metabolism, because this has been linked to insulin resistance in numerous studies [6–11]. This is thought to occur through accumulation of nonesterified intermediates from lipid metabolism that inhibit insulin signaling [12, 13], which is referred to as lipotoxicity [6]. Thus, lipotoxicity can develop from increased uptake of fatty acids (FA), decreased synthesis of FA and diacylglycerol (DAG) into triacyglycerol (TAG), and/or increased release of FA from TAG, which will all lead to increased DAG levels and impaired glucose tolerance. Another hypothesis suggests that impaired mitochondrial function and lipid oxidation lead to accumulation of lipid intermediates (DAG and ceramides) and insulin resistance [14]. In the light of the study by Phillips et al. [4] and the preclinical data by Langhi et al. [5], the question arises as to whether chronic simvastatin use changes fatty acid storage and oxidation in humans. The literature on the effect of statins on FA oxidation rate in humans is conflicting with studies reporting either no effect [15, 16] or simvastatin-induced impaired fat oxidation [17–20]. One explanation for controversy could be that the studies that report no effect of statins on fat oxidation (FO) only use treatments of shorter duration (5 days to 8 weeks) [15, 16]. Interestingly, one study investigated lipid oxidation in myotubes from statin-treated patients and control participants and found that octanoate and palmitate oxidation was increased in myotubes from patients, and this was concurrent with a reduced fat oxidation at rest in the patients (measured with indirect calorimetry) [21]. This indicates that measurement of lipid oxidation at the skeletal muscle level might not be comparable to whole body fat oxidation measurements.

The aim of this study was to explore whether there is a relationship between simvastatin-induced insulin resistance and skeletal muscle lipid metabolism alterations, which may be linked to lipotoxicity. We have in a previous study reported changes in mitochondrial function in the participants included in the present study [22]. Our hypothesis was that chronic simvastatin treatment (>1 year) leads to impaired skeletal muscle lipid metabolism, as a result of attenuated protein expression of the major proteins involved in lipid uptake (i.e., lipoprotein lipase (LPL), endothelial lipase (EL), CD36, and plasma membrane-bound fatty acid-binding protein (FAPBpm)), lipid synthesis (i.e., DGAT1 and 2), lipid droplet regulation (i.e., perilipins 2, 3, and 5), and/or lipolysis (i.e., adipose triacylglycerol lipase (ATGL) and hormone-sensitive lipase (HSL)).

## 2. Materials and Methods

### 2.1. Subjects

Twenty men were recruited to participate in the study; ten patients with hypercholesterolemia treated with simvastatin for at least 1 year (1–5 years, *n* = 7; 5<, *n* = 3) were treated with the following doses (10–40 mg/day; 10 mg/day, *n* = 1; 20 mg/day, *n* = 4; 40 mg/day, *n* = 5) and ten healthy control subjects. The groups were matched for age, weight, BMI, body fat (both total and abdominal fat percentage), and maximal oxygen uptake (VO_2max_) (Table 1). Familial predisposition for type 2 diabetes, signs of coronary ischaemia, and other medication than simvastatin was an exclusion criterion. A prior manuscript that focused on the effect of simvastatin on muscle mitochondrial respiration in the subjects included in the present study has already been published [22]. After the subjects were recruited and blood and skeletal muscle were analyzed, it appeared that one of the men in the control group had impaired glucose tolerance and data from this subject has therefore been omitted. The ethics committee of the municipality of Copenhagen and Frederiksberg in Denmark approved the study protocol (H-4-2009-095), and oral and written consents were obtained from each participant in accordance with the Helsinki Declaration.

### 2.2. Experimental Protocol

The experiment has previously been described in full detail [22]. In short, the men reported to the laboratory in the morning after an overnight fast (>10 h) on a screening day and an experimental day. On the screening day, a standard 2 h oral glucose tolerance test (OGTT) was performed. Moreover, a resting ECG was recorded and a dual-energy X-ray absorptiometry (DXA) scan (Lunar Prodigy Advance, Lunar, Madison, WI, USA) was performed to access body composition. Finally, an incremental cycling test was performed to determine maximal oxygen uptake (VO_2max_) (Jaeger ER 800; Würzburg, Germany). On the experimental day, subjects were placed in a supine position for 30–40 min and resting metabolic rate (RMR) was measured by ventilated hood (Oxycon Pro; Jaeger; Würzburg, Germany). After this, a blood sample was drawn and a muscle biopsy obtained from the m. *vastus lateralis* using the Bergström needle biopsy technique with suction [23]. One part of the muscle was rapidly frozen for Western blotting and stored at −80°C for later analysis. The subjects were asked to rest for 30 min before an incremental cycling test was performed to determine maximal fat oxidation (MFO) and the intensity where MFO occurred (FatMax), as described in detail elsewhere [24]. This protocol has previously been validated in obese untrained subjects [25].

### 2.3. Blood Analysis

Plasma adiponectin and leptin concentrations were measured using specific high-sensitive human ELISA kits. The adiponectin assay (Linco Research, St. Charles, MO, USA) had an intra-assay coefficient of variation of 3.9%. The leptin assay (R&D Systems, Minneapolis, MN, USA) had an intra-assay coefficient of variation of 3.2%.

### 2.4. Western Blotting

Approximately 25 mg muscle tissue was homogenized in cold buffer with protease and phosphate inhibitors added (50 mM Tris pH 8.0, 150 NaCl, 1% NP-40, 0.5% Na-deoxycholate, 0.1% SDS, 2.5 mM PMSF, 20 mM *β*-glycerophosphate, 10 mM pyrophosphate, and 2 mM sodium orthovanadate), including a mini-EDTA protease inhibitor tablet according to the instructions of the manufacturer (Roche Diagnostics, Mannheim, Germany). Homogenization was done at 30 Hz for 2 × 2 min at −20°C in a TissueLyser (Qiagen Retsch, Haan, Germany) or until the sample was completely dissolved. Thereafter, the homogenate was sonicated for 2 × 5 sec. Protein concentration was measured by bicinchoninic acid assay (Pierce, Rockford, IL, USA) in triplicate, and a maximal coefficient of variation of 5% between replicates was accepted.

For all muscle samples, an equal amount of protein (20 *μ*g) was heated to 95°C for 10 minutes and electrophoresed in either 12.5% or 4–15% polyacrylamide sodium dodecyl sulphate gels (26-well 2-amino-2-(hydroxymethyl)-1,3-propandiol (Tris)-HCl precast gel, Criterion, Bio-Rad, Copenhagen, Denmark) and electrotransferred to a PVDF membrane (0.2 *μ*m pores, Bio-Rad, Copenhagen, Denmark). The membranes were blocked for 1–2 hours at room temperature with either skimmed milk (Merck, Darmstadt, Germany) or bovine serum albumin (BSA, Fraction V Modified Cohn) and diluted in Tris-buffered saline (10 mM Tris base, 150 mM NaCl, pH 7.4) ± 0.05% Tween 20. The membranes were then incubated with the primary antibody overnight at 4°C. The primary antibodies were anti-ATGL (Ab109251, Abcam, Cambridge, UK, ~55 kDa), anti-CD36 (Ab17044, Abcam, Cambridge, UK, ~80 kDa), anti-FABPpm (GOT2, Ab93928, Abcam, Cambridge, UK, ~42 kDa), anti-HSL (G7, sc-74489, Santa Cruz Biotechnology Inc., Heidelberg, Germany, ~80 kDa), anti-LPL (H53, sc-32885, Santa Cruz Biotechnology Inc., Heidelberg, Germany, ~80 kDa), anti-perilipin 5 (Novus Biologicals, Littleton, CO, USA, ~50 kDa), anti-perilipin 3 (Sigma, Prestige Antibodies, St. Louis, MO, USA, ~47 kDa), anti-perilipin 2 (Novus Biologicals, Littleton, CO, USA, ~50 kDa), anti DGAT1, (NB100-57086, Novus Biologicals, Littleton, CO, USA ~55 kDa), and anti-DGAT2 (Ab96094, Abcam, Cambridge, UK, ~44 kDa). The membranes were washed in Tris-buffered saline ± 0.05% Tween 20. Secondary antibodies were Goat anti-rabbit horseradish peroxidase conjugated and goat anti-mouse horseradish peroxidase conjugated (Dako, Glostrup, Denmark). Blots were developed in ECL detection reagents (GE Healthcare, Little Chalfont, UK), and the chemiluminescence emitted from immune complexes was visualized with a LAS 4000 image analyzer (GE Healthcare, Little Chalfont, UK). The images of the membranes and Coomassie stained gels were quantified by ImageQuant TL software version 7.0 (GE Healthcare, Little Chalfont, UK).

In order to check for even transfer throughout the membranes, a homogenate was loaded on each gel on several lanes dispersed over the gel. This homogenate was made from a mix of biopsies from 2 subjects that were matched by age, VO_2max_, and fat-free mass and handled as the other samples.

In order to control equal protein loading and also transfer efficacy from gels to membranes, all our gels were stained with Coomassie Blue (Pierce, Rockford, IL, USA).

### 2.5. Statistics and Calculations

Data are presented as means ± SE in the text and in all figures and tables. *P* < 0.05 was considered significant. Differences between the two groups were evaluated with Student's *t*-test. All statistical analyses were performed in Sigma Plot 12.5 (Systat Software Inc., San Jose, USA). Pearson's correlation analysis was used to establish the presence of correlations. Whole-body fat oxidation was calculated from VO_2_ and VCO_2_ values during the last 60 s of each exercise step in the graded exercise tests, using standard indirect calorimetry equations [26]. For each subject, polynomial curve fitting (Sigma Plot 12.5, Systat Software Inc., San Jose, USA) was used to determine whole-body peak fat oxidation. For determination of fat oxidation at rest, data obtained during the hood measurements were used. The Cederholm index for glucose homeostasis was calculated as previously described [27]; briefly, glucose and insulin concentrations during the oral glucose tolerance test, body weight, and the amount of glucose ingested were taken into account when the index was calculated.

## 3. Results

### 3.1. Subject Characteristics

The primary characteristics of the groups have been published previously but are also included here [22]. The 10 patients in treatment with simvastatin had signs of lower insulin sensitivity compared with the 9 control subjects, calculated by the Cederholm index and indicated by a higher HbA1c than in the controls [22]. Apart from that, there were no differences between the groups in age, weight, BMI, body fat percentage, and VO_2max_ (Table 1). Neither were there any differences in the plasma high-density lipoprotein (HDL), low-density lipoprotein (LDL), nonesterified fatty acids (NEFA), or triglycerides (Table 1). Adiponectin and leptin were comparable between the two groups (Table 1). Furthermore, no difference was seen in resting fat oxidation, MFO, or FatMax between the groups (Table 2).

### 3.2. Muscle Characteristics

**Figure 1:**
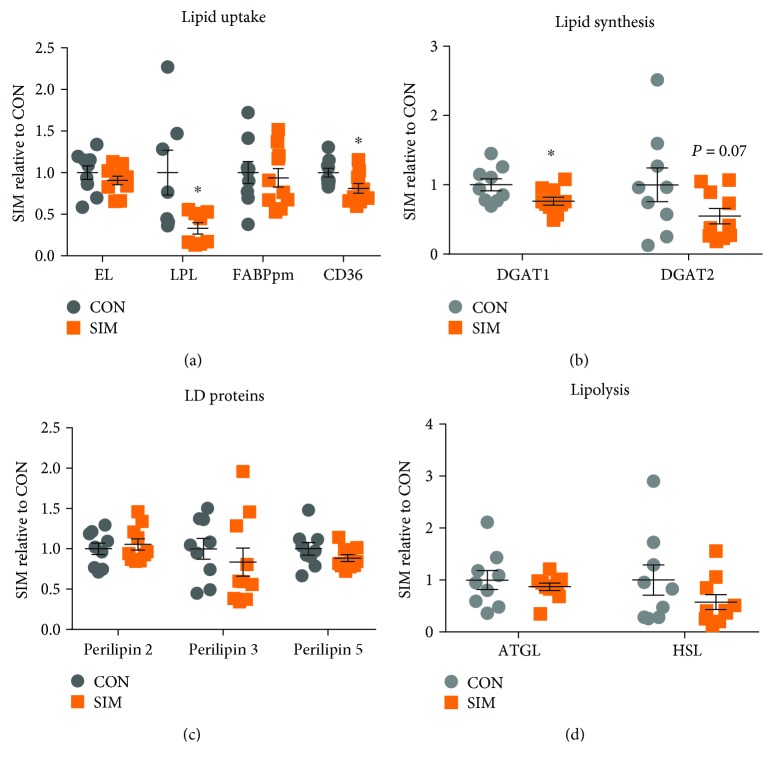
Protein expression of major proteins in skeletal muscle from patients in treatment with simvastatin (yellow squares) and matched controls (grey circles). (a) Lipid uptake-related proteins EL (endothelial lipase), LPL (lipoprotein lipase), FAPBpm (plasma membrane-bound fatty acid-binding protein), and CD36. (b) Lipid synthesis-related protein DGAT 1 and 2 (diacylglycerol acyltransferase). (c) Lipid droplet regulation, perilipins 2, 3, and 5. (d) Lipolysis: ATGL (adipose triacylglycerol lipase) and HSL (hormone-sensitive lipase). Data are mean ± SE, ^∗^*P* < 0.05. Data are presented as relative to mean of the control. Representative blots are shown in Figure 2.

**Figure 2:**
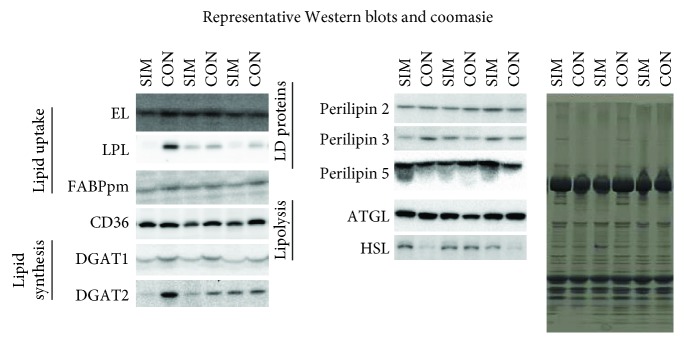
Representative Western blots for Figure 1 for patients in simvastatin treatment (SIM) and matched controls (CON). ATGL: adipose triglyceride lipase; DGAT: diacylglycerol acyltransferase; EL: endothelial lipase; FABPpm: plasma membrane-bound fatty acid-binding protein; HSL: hormone-sensitive lipase; LPL: lipoprotein lipase; perilipins 2, 3, and 5. Representative picture of a coomassie staining that visualizes equal protein loading.

Patients in treatment with simvastatin had lower skeletal muscle CD36, LPL, DGAT1, and a tendency towards lower DGAT2 (*P* = 0.07) expression compared to the control group (Figures 1(a) and 1(b)). No significant differences were observed in the expression level of EL, FAPBpm, perilipins 2, 3, and 5, ATGL, and HSL (Figures 1(a), 1(c), and 1(d)). Representative blots for Figure 1 is given in Figure 2. When correlations between glucose tolerance (Cederholm index) and the measured proteins were made, some tendencies were observed (DGAT1: *P* = 0.06; *r* = 0.47; DGAT2: *P* = 0.08, *r* = 0.43).

## 4. Discussion

The major finding of this study is that chronic simvastatin-treated patients with impaired glucose homeostasis had a lower muscle capacity to recruit exogenous FA (decreased CD36 and LPL expression) and a lower capacity to synthesize intramuscular FA and DAG into TAG (lower DGAT1 and a tendency to lower DGAT2) compared to controls matched for age, weight, BMI, body fat, and maximal oxygen uptake. It is possible that this difference may lead to intracellular accumulation of FA and DAG, which have lipotoxic properties. The results add knowledge to our understanding of the molecular mechanism behind attenuated insulin sensitivity induced by yearlong chronic use of simvastatin.

The lower CD36 and LPL expression in the simvastatin-treated patients (Figure 1(a)) was somewhat unexpected as this indicates an attenuated capacity to incorporate exogenous fatty acids, which would indicate a reduced risk of lipotoxic intermediate accumulation. It has previously been reported that insulin resistance was linked to increased protein expression of CD36 [28]. Two main steps regulate cell FA uptake, plasma FA concentration, and FA transporters at the cell membrane. In addition, the plasma lipoprotein profile may also influence the membrane transport of FA [27]. However, there was no apparent difference in plasma lipoprotein profile between the groups (Table 1), but it is still possible that subfractions of HDL and LDL that we did not measure may have differed and thus have induced the difference in FA transporters.

Even though it was not stated in our hypothesis, our finding of lower CD36 in the simvastatin-treated patients is interesting, because Anderson et al. recently reported an important role for CD36 in coenzyme Q10 (Q10) uptake in brown adipose tissue from CD36 KO mice [29]. Thus, the decreased CD36 could explain the decreased Q10 that we previously have reported in the simvastatin-treated men [22]. In skeletal muscle of patients in treatment with simvastatin, decreased Q10 have been associated with myalgia [30]. Thus, our observation elucidate a possible mechanism through which simvastatin may reduce muscle Q10 and hence increase risk of myalgia.

The expression of proteins related to lipid droplet storage/regulation (perilipins 2, 3, and 5) and lipolysis (ATGL and HSL) did not differ between the groups, and this is concomitant with the similar whole-body fat oxidation. To our knowledge, protein expression of lipid droplet storage/regulation and lipases has never been investigated in human skeletal muscle exposed to chronic simvastatin treatment before. However, incubation of mouse and human primary hepatocytes with statins for 48 hours leads to no changes in perilipins 2 and 3 but did induce a reduced perilipin 5 expression (protein and mRNA) [5]. In human skeletal muscle, perilipin 5 has been linked to fatty acid oxidation and has been associated with a higher capacity to release FA for oxidation possibly by linking interaction between lipid droplets and mitochondria [31, 32]. Furthermore, Phillips et al. reported larger lipid droplets and IMTG accumulation in skeletal muscle with statin treatment [4]. Thus, a difference in perilipin 2 between the groups could have been expected, because perilipin 2 and IMTG levels have been shown to correlate in some (measured by Oil Red O) [33–35], but not all (measured by electron microscopy) [36] studies. It could be speculated that some of the changes seen in the proteins measured in the present study could be due to changes in fiber-type distribution, and in the same subjects, small changes have been reported in MHC content [22].

Insulin resistance has previously been linked to a reduced whole-body fat oxidation [37], but consensus has not been reached [38, 39]. Since statin treatment leads to insulin resistance, we investigated maximal whole-body fat oxidation at rest and during exercise. Adiponectin and leptin were comparable between the groups despite differences in glucose tolerance (Cederholm index). In the present study, the fat oxidation rate was similar between simvastatin-treated patients and control subjects. This is in line with the studies of short treatment (5 days–8 weeks) [15, 16], but not those of longer treatment [17–19]. However, in the long-term studies, it is possible that a different plasma lipid profile [17, 19], VO_2_max, or age [18] between the treatment and control groups may have caused the reported difference in fat oxidation rate. A strength of this study is that the groups were matched for these parameters.

It is a paradox that simvastatin treatment lowers cholesterol concentration and hence decreases CVD risk but conversely also increases the risk of insulin resistance. It is well known that CVD and decreased insulin sensitivity have deep ramifications in each other's pathology. Furthermore, decreased insulin sensitivity is a critical factor which accelerates independent risk factors of CVD including hypertension, obesity, and dyslipidemia.

### 4.1. Limitations

A cross-sectional study design makes it difficult to distinguish whether simvastatin is the direct cause of the observed differences or it is secondary to other factors (i.e., impaired glucose homeostasis and/or genetic factors), yet the two groups were well matched. On the other hand, a strength of the study design is that we can evaluate a yearlong chronic use of simvastatin in patients with hypercholesterolemia. Longitudinal studies in patients with hypercholesterolemia and matched controls would give further insight into these mechanisms.

## 5. Conclusion

A known adverse effect of simvastatin treatment is impaired glucose homeostasis. Here, we show that this is linked to a decreased capacity to incorporate exogenous FA and to a lower intramuscular capacity to synthesize TAG in lipid droplets. Overall, this muscle adaptation may lead to increased cellular levels of DAG, a lipotoxic intermediate that may inhibit insulin signaling and hence induce insulin resistance.

## Figures and Tables

**Table 1:** Subject characteristics.

	Patients (*n* = 10)	Controls (*n* = 9)
Age (years)	45 ± 2	45 ± 1
Treatment time (years)	5 ± 1	—
Weight (kg)	93 ± 4	91 ± 4
BMI (kg/m^2^)	27 ± 1	27 ± 1
Body fat (%)	28 ± 2	27 ± 2
LBM (kg)	63 ± 2	63 ± 2
VO_2max_ (ml O_2_/min/kg BW)	38 ± 1	40 ± 2
IPAQ (kcal/week)	3455 ± 672	3128 ± 709
HbA1c (%)	5.7 ± 0.1^∗^	5.2 ± 0.1
NEFA (*μ*mol/l)	455 ± 59	412 ± 100
TG (mmol/l)	1.4 ± 0.2	1.3 ± 0.2
Cholesterol (mmol/l)	4.8 ± 0.3	4.3 ± 0.2
LDL (mmol/l)	3.1 ± 0.3	2.7 ± 0.2
HDL (mmol/l)	1.3 ± 0.1	1.2 ± 0.1
Adiponectin (ng/ml)	6004 ± 503	5065 ± 637
Leptin (pg/ml)	4411 ± 750	4139 ± 859
SI (mg·l^2^/mmol·mU·min)	39 ± 6^∗^	54 ± 4

Data are mean ± SE. Abbreviations: BW, body weight; HbA1c, glycated hemoglobin; HDL, high-density lipoprotein; IPAQ, International Physical Activity Questionnaire; LBM, lean body mass; LDL, low-density lipoprotein; NEFA, nonesterified fatty acids; SI, peripheral insulin sensitivity (Cederholm index); TG, triglycerides; VO_2max_, maximal oxygen uptake. ^∗^*P* ≤ 0.05. Data has previously been published [22].

**Table 2:** Fat oxidation at rest and during exercise.

	Patients (*n* = 10)	Controls (*n* = 9)
	Rest	
FO_rest_ (g/min)	0.08 ± 0.01	0.06 ± 0.01
	Exercise	
MFO (g/min)	0.30 ± 0.03	0.29 ± 0.04
FatMax (% of VO_2MAX_)	39 ± 2	39 ± 2

Data are mean ± SE. Abbreviations: FatMax, intensity where MFO occurs; FO, fat oxidation; MFO, maximal fat oxidation; VO_2max_, maximal oxygen uptake.

## Data Availability

The data related to the present manuscript could be available from the corresponding author upon request.
